# Artichoke Cultivars (var. “Blanca de Tudela”) under Elevated Ozone Concentrations

**DOI:** 10.1100/tsw.2002.150

**Published:** 2002-03-26

**Authors:** M.J. Sanz, J.L. Porcuna, E. Calvo, C. Martin

**Affiliations:** Fundación Centro de Estudios Ambientales del Mediterráneo (CEAM), Parc Tecnològic, C/C.R. Darwin, 14. 46980 Paterna, Valencia, Spain; Servicio de Protección a los Vegetales, Consellería de Agricultura Pesca y Alimentación, Pista de Silla, s/n. Valencia, Spain

**Keywords:** resprouting, ozone, Mediterranean crops, artichoke

## Abstract

Ozone concentrations rise to phytotoxic levels from spring to autumn at western Mediterranean basin coastal sites, where artichoke is one of the most important crops. Simultaneously, from year to year and especially since the early 1980s, resprouting of the stumps has been decreasing in Valencian Community artichoke plantations. To see if ozone might be playing a role in this decrease, a number of plants were exposed to different levels of ozone. Results of the ozone treatments showed reduced biomass in the offshoots of plants exposed to the highest ozone treatment. The exposure to ambient ozone during the stump-establishment period, when compared to filtered-air conditions, resulted in a reduction in yield when plants were transplanted in the field under ambient ozone concentrations. And when plants were exposed to acute short picks, typical ozone visual injury appeared in the older leaves.

## INTRODUCTION

The concentrations of tropospheric ozone in the Mediterranean basin have increased in the last decades, especially since the 1980s, reaching levels that can be considered phytotoxic[1]. Parallel to this increase, damages produced by this pollutant have been reported on crops (such as tomato, watermelon, muskmelon, peanuts, potato, and tobacco) on the east coast of Spain[2,3,4,5] and Italy[6].

Artichoke (*Cynara scolymus* L.) is a native of the Mediterranean. It is a perennial in the thistle group of the sunflower (Compositae) family. At full growth, the plant spreads to cover an area of about 6 ft in diameter and reaches a height of 3 to 4 ft. Its long, arching, deeply serrated leaves give the plant a fern-like appearance. It is now grown in many countries of the world, but about 90% of the acreage is found in the Mediterranean area, with Italy being the largest producer

(468,000 t, nearly 35% of the world production); Spain and France rank second (444,000 t) and third (93,000 t), respectively[7]. In the 1970s, up to 67% of the Spanish artichoke production came from the Valencian Community, whereas by the mid 1980s, this percentage had decreased to 37% of the total production[8].

In Spain the main propagation method for planting artichokes is with root sections attached to basal stem pieces; seed propagation is less used. These cuttings, which are often referred to as “stumps” or “offshoots”, are obtained from established fields scheduled for replanting. Artichoke fields are maintained in perennial culture for 5 to 10 years. Each cropping cycle is initiated by “cutting back” the tops of the plants to several inches below the soil surface to stimulate development of new shoots. In most of the plantations on the east coast of Spain, growth starts in August, harvest begins around mid-November and continues until mid-May, and plants are cut in mid-June[8]. It is well known that artichoke plants require cool temperatures for the production of the fleshy, nonfibrous flower heads[9]; thus, production finishes before warm temperatures appear in Mediterranean climates (before May–June in the Valencian region).

In the western Mediterranean basin, ozone concentrations rise up to phytotoxic levels from spring to autumn at the coastal sites where artichokes are cultivated[10]. For the last 2 decades, agricultural technicians from the Protection and Certification Department of the Valencia Ministry of Agriculture have reported anomalous behavior in the successive generation resprouting of artichoke material planted on the eastern coastal plains of the Valencian Community; they have seen that more and more offshoots were not surviving the successive plantations. Since they could not find an explanation related to pathological or cultural problems alone, they classified the phenomenon as an anomalous early degeneration (aging) of the material. Recently, it has been hypothesized that ozone may contribute to this observed early degeneration of the propagation material.

The objective of the present paper is to determine whether or not ozone affects the artichoke by reducing the vitality of the material to be used the following year, and whether the observed degeneration is related to the high ozone concentrations in the geographic area where the phenomenon is observed.

## METHODOLOGY

Offshoots (n = 216) of “Blanca de Tudela” artichoke plants of similar weight from Navarra (Spain) were planted[11] in 6-l pots containing a mixture of sand and peat (2:1) at the end of July 1996. The offshoots were weighed at the beginning (air dry weight is presented). Offshoots were previously treated with Monocut 50 ppm (a systemic fungicide) to prevent *Rhizoctonia solani* fungal attacks.

During 1996–1997, ozone exposure experiments were conducted in six open-top chambers (OTCs)[12] located on the Valencia Plain (Benifaió: 0º24´48´´W, 39º16´43´´N) near an artichoke-intensive cultivation area described elsewhere[13]. The experimental field is located in La Ribera, an area very well known for its artichoke production[8]. The concentrations of $O_3$, NO, and $NO_2$ in each chamber were measured continuously with two monitors (nitrogenous oxides monitored with a DASIBI 2108 and ozone with a DASIBI 1008 RS). During the experiment, irrigation was provided on a daily basis using a drip-irrigation system with a 1:100 mixture containing 15 g $l^{-1}$ of monoammonium phosphate, potassic nitrate, and ammonium nitrate.

Applied treatments were: filtered (using charcoal filters) and nonfiltered air (with short and acute discontinuous ozone fumigations in late spring in the nonfiltered chambers; see Table 1 for concentrations).

In mid-September, the nonsuccessful (dead) offshoots were counted, and 10 randomly selected successful offshoots (at least three leaves present, n = 60) per chamber were transplanted to 30-l pots containing a mixture of sand and peat (2:1). The reason for the selection of 10 individuals for the second phase of the OTC exposures was related to the space needed for adult plants and the limited space in the chamber. Leaves and shoots were cut at the end of the experiment (air dry weight is presented).

The remaining offshoots (n = 87, not used for the OTC exposure, second phase) were transplanted to the field from the 6-l pots, and regular management was followed[13,14]. Before plantation, the field was prepared with 6 kg of compost for each lineal meter. During the field experiment, irrigation was provided by a dripping system once a day. Artichokes were collected and weighed once a week. The field plot was located next to the OTC experimental field.

Statistical analyses were performed with SPSS for Windows (SPSS, Inc.); one-way ANOVA was used to compare the offshoot weights after OTC exposures, and a t-test was used to compare the artichoke production in the field ($p < 0.05$) in order to note any significant differences.

## RESULTS AND DISCUSSION

Ozone exposures during the experiment are presented as an AOT40 index[15] that has been calculated as the accumulated 1-h averages over 40 ppb when total radiation was >50 $W/m^2$ during the exposure time in the OTCs. Exposures (Table 1) at the end of the experiment were above the critical level proposed by the UN-ECE for the ozone-treated chambers[15], but were not unusual for the east coast of Spain[16].

The plant establishment period was apparently not affected by ozone; 39% of the offshoots remained nonrooted with equal distributions among treatments. Potted offshoots had similar weights at the beginning of the experiment.

In Fig. 1, where only the weights of the 60 offshoots selected for the whole period are considered, initial weight is similar for both of the treatments (filtered air vs. nonfiltered air plus acute short picks, while at the end of the experiment the offshoots, after cutting the leaves and shoots, were significantly bigger in the plants grown under filtered-air conditions. It is known that ozone suppresses root growth more than shoot growth. Moreover, increases in allocations to shoots with respect to roots have been attributed to metabolic costs to repair stress damage[17].

**TABLE 1**
**Average Hourly Maximum Ozone Concentration (ppb) and AOT40 Index (ppb.h) during the Different OTC Exposition Steps for Each Treatment and the AOT40 Accumulated at the End of the Experiment for Each Treatment (n = 3)**

| | Filtered | | | Nonfiltered + $O_3$ | | | |
|---|---|---|---|---|---|---|---|
| Date | Average (ppb) | Max (ppb) | AOT40 (ppb.h) | Average (ppb) | Max (ppb) | AOT40 (ppb.h) | No. of Days |
| 1/8/96 to 18/9/96* | 8.5 | 49.7 | 36.7 | 27.1 | 67.7 | 2,161.0 | 48 |
| 19/9/96 to 12/5/97 | 10.2 | 53.0 | 184.7 | 19.5 | 70.0 | 5,262.0 | 234 |
| 13/5/97 to 16/5/97[a] | 13.9 | 39.7 | 0.0 | 48.9 | 134.0 | 1,988.0 | 4 |
| 17/5/97 to 27/5/97 | 16.7 | 51.7 | 33.7 | 32.0 | 64.0 | 878.7 | 11 |
| 28/05/97[b] | 12.2 | 35.3 | 9.7 | 52.6 | 154.0 | 684.0 | 1 |
| Total accumulated | | | 264.7 | | | 10,974.0 | 298 |

Note: Additions of ozone were for 6 h/day, from 9 a.m. to 3 p.m.

* Plants in 6-l pots, establishment period. On the other dates, plants in 30-l pots.
[a] Addition of 90 ppb of ozone.
[b] Addition of 120 ppb of ozone.

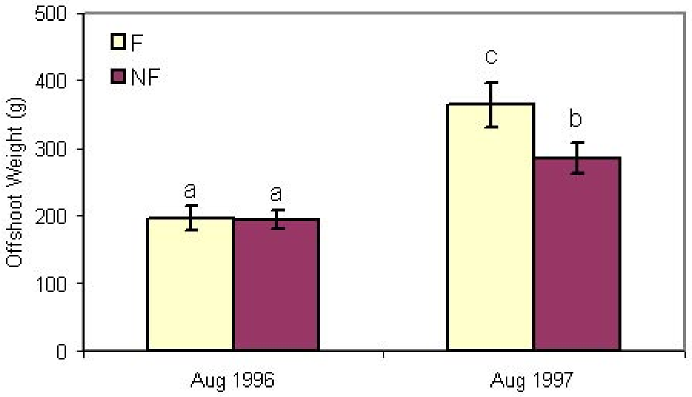

**FIGURE 1**. Offshoot air dry weight (g) before planting and at the end of the experiment, grown under filtered and nonfiltered/fumigated conditions (see Table 1 for exposures). One-way ANOVA, different letters indicate significant differences ($p < 0.05$).

Thus, the outcome often seems to be the preferential translocation of assimilate to shoot tissues or retention in the shoot under chronic ozone stress[18]. The small size of the offshoots grown under relatively high ozone levels may be due to a deficient carbon allocation to the stumps. And this may later result in lower success in the next growing season, since plant resprouting depends on the reserves in the stumps.

The yield from the potted plants was low and the artichokes were small; all of them were classified as noncommercial[19]. In contrast, the plants transplanted to the field produced up to 0.6-kg/plant commercial heads, in the range of the reported production for the region[8]. Differences in production in the field were found among treatments (Fig. 2) in total production (kg per treatment) and in mean individual plant production (kg per individual plant). Artichoke plants coming from the filtered-air treatment produced more (an average of 0.56 kg plant$^{-1}$) than the plants coming from nonfiltered air (an average of 0.40 kg plant$^{-1}$), 11.2 and 8 Mt ha$^{-1}$, respectively. Thus, reducing ozone concentrations to low levels during the establishing period of the plant, until the plant has at least three leaves, results in an increase in production when compared with plants that were established at ambient concentrations. The results suggest that the first growth stages are affected by the ambient ozone concentration and that very low ozone concentrations may prevent production reductions during the first year after planting new stumps.

In the OTCs, after the first fumigation episode, older artichoke leaves showed characteristic ozone symptoms (Fig. 3C); they only appeared in the nonfiltered plus ozone treatment as an upper leaf surface interveinal stipple, and they were not seen in the field plot. The observed stipple is shown as the discoloration of small groups of cells between the veins, appearing as uniformly-sized red-to-brown spots that give a general reddish color to the upper leaf (Fig. 3A), leaving the veins green (Fig. 3B), when compared with noninjured leaves (Figs. 3D and 3E). Shaded parts of the leaves either did not show the stipple or showed it with less intensity than the sun-exposed parts, as reported for typical ozone injury[20]. The appearance of visible injury on the leaves suggests that the repair capacity of the plant is surpassed by the short high peaks of ozone exposure. Visible injury results in many cases from an acute ozone dose[21], whereas chronic exposure causes a reduction in growth and changes in carbon allocation[18]. Similar foliar symptoms have been observed in field surveys performed in rural areas of the Turia Valley (Valencia) where phytotoxic ozone levels have been recorded (J.L. Porcuna, personal communication). Such ozone-like symptoms in the field have been reported with weather conditions that favor photochemical processes and coincide with recordings of high ozone concen-

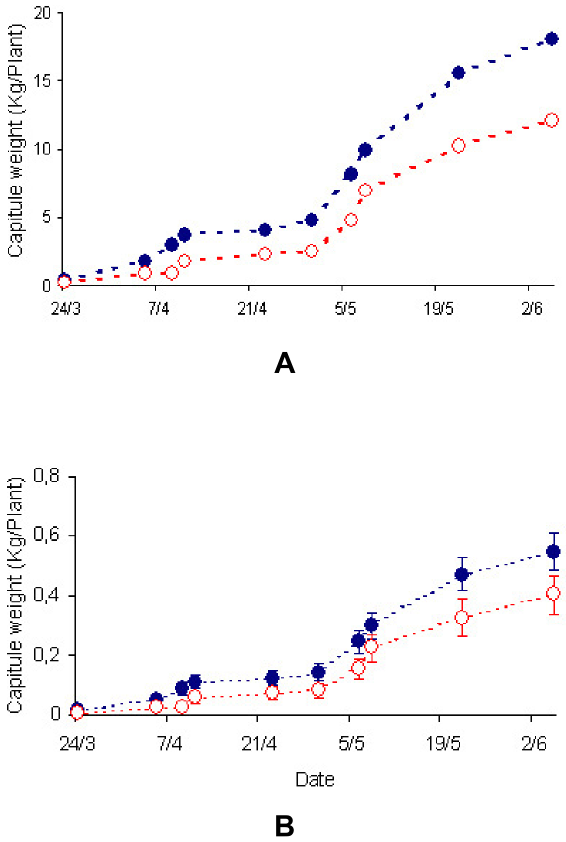

**FIGURE 2**. (A) Total production in the field (kg per treatment). (B) Mean and standard error of individual plant production. Blue circles indicate artichoke plants coming from charcoal-filtered air; red circles indicate artichoke plants coming from nonfiltered air. No statistically significant differences were found; one-way ANOVA ($p < 0.05$).

trations in the Air Quality Network in the area[22]. With respect to the spatial distribution, visible symptoms have been observed in areas where high ozone peaks had recently been recorded during measurement campaigns in late spring (up to 130-ppb hourly peaks, unpublished data). Taking into account our results and the field symptoms observed, we suspect that visible injury appears after acute peaks and may not be the result of chronic exposures. Further research is needed to better understand the effects of high peaks vs. chronic exposures in artichokes; nonetheless, present results suggest that chronic exposures result in reductions of growth and yield, whereas peaks cause visible foliar injury.

No information about the sensitivity of artichoke to ozone has been found in the scientific literature except an unclear citation of artichoke as an ozone-sensitive crop in a UN-ECE report[23]. The results presented here suggest that the “Blanca de Tudela” artichoke may be sensitive to ambient ozone concentrations. When ozone concentrations increase, the spring artichoke head production is almost finished; however, before the leaves dry out in June–July, ozone may affect the accumulation of reserves for the following crop as well as the sprouting of new leaves in late summer. This could explain the fact that from year to year, resprouting of the offshoots planted in previous years has been decreasing in Valencian Community plantations, especially since the late 1980s.

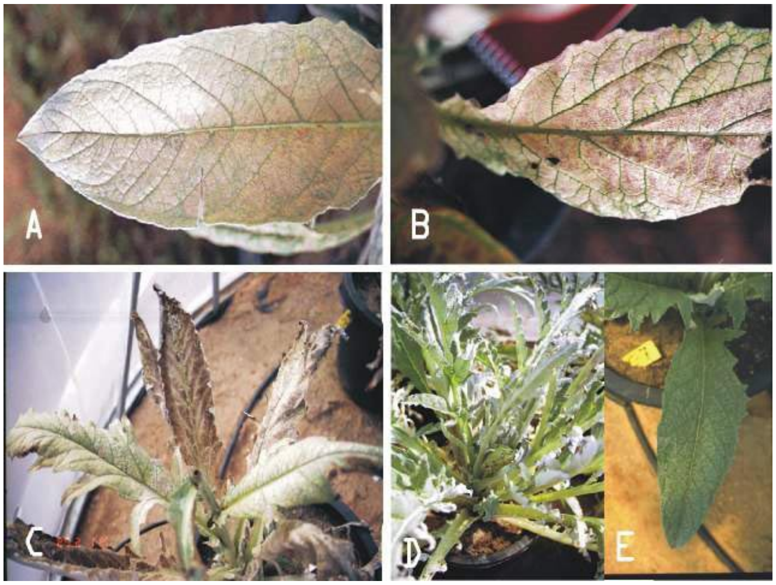

**FIGURE 3**. (A) Artichoke leaf with developing visual ozone injury; symptoms appeared in the upper part of older leaves. (B) Leaf strongly affected. (C) General view of fumigated plant. (D) Control.

## ACKNOWLEDGEMENTS

The research team is supported economically by the Generalitat Valenciana (Consellería de Medio Ambiente and Consellería de Agricultura, Alimentación y Pesca) and Bancaixa. The work presented here has been financed by the projects GV-C-AG-01-108-96 and ASS98-06-13, CEAM and SSCV of Consellería de Agricultura, Pesca y Alimentación. We would also like to thank Dr. C. Jordá for her advice on issues related to pathology and crop management.
